# Osteogenesis imperfecta type I: A case report

**DOI:** 10.3892/etm.2014.1653

**Published:** 2014-03-31

**Authors:** JIANMIN REN, XIAOJIE XU, XIANGDONG JIAN, JIERU WANG

**Affiliations:** 1 Department of Endocrine and Metabolic Diseases, Qilu Hospital, Shandong University, Jinan, Shandong 250012, P.R. China; 2 Department of Poisoning and Occupational Diseases, Qilu Hospital, Shandong University, Jinan, Shandong 250012, P.R. China

**Keywords:** osteogenesis imperfecta type I, heterogeneous disease, endocrine

## Abstract

A 15-year-old male patient was admitted to hospital having experienced repeated fractures over the previous three years, predominantly due to falling down or overexertion. The clinical signs and radiological features, such as recurrent fractures, blue sclera and low bone mineral density (BMD) level, all led to the diagnosis of a mild form of osteogenesis imperfecta (OI) type I. The patient began treatment with a regular intake of calcium (1,000 mg/day), an adequate intake of vitamin D (800 U/day) and intravenous pamidronate (60 mg). Following four months of treatment, the symptoms and quality of life of the patient improved. This patient appears to be a rare case of OI type I.

## Introduction

Osteogenesis imperfecta (OI) is a genetic disorder that is characterized by recurrent fractures, low bone mass, blue sclera and dentinogenesis imperfecta (DI). It is a rare disorder with an overall incidence of ~1 in 10,000–20,000 births (1). The etiology remains unclear; however, it is estimated that ~90% of cases are associated with mutations in the collagen type I, α1 (COL1A1) or COL1A2 genes and the remaining 10% of cases are associated with other genes (2). OI is a heterogeneous disease and type I is the most common and mild form (1). At present, there is no effective therapy. The present report presents the case of a 15-year-old Chinese male with OI type I. Informed consent was obtained from the patient. The Ethics Committee of Shandong University (Jinan, China) approved the study.

## Case report

A 15-year-old male was admitted to Qilu Hospital (Jinan, China) complaining of repeated fractures over the previous three years, predominantly due to falling over or overexertion. The fractures occurred approximately every 3–4 months, often in the forearms and legs. The fractures healed with no significant delay when treated with external fixations. The patient did not experience bone pain, limb and joint deformities or muscle weakness.

One year previously, the patient had been prescribed a six-month treatment of Caltrate and calcitriol (one of each tablet per day); during this period the patient experienced one bone fracture. However, he suffered from two further fractures as a result of minor impacts following discontinuation of the treatment over the subsequent six months. The patient had not taken other medications, such as steroids, which affect bone metabolism. The patient received an appendectomy in 2006 due to acute appendicitis and recovered well following the surgery.

The patient was born following a full-term pregnancy by spontaneous vaginal delivery without fetal distress or asphyxia: Birth height, 50 cm (50th percentile) and birth weight, 3,500 g (50th percentile). The patient was breastfed with no delayed eruption of milk or permanent teeth, and was able to sit at eight months and walk independently at 16 months. At 8 and 12 months of age, the patient presented with episodes of nondisplaced fractures resulting from minor impacts. The patient grew more slowly than his peers (with regard to height) until age eight years, but exhibited normal intellectual development. The parents were non-consanguineous and healthy and there was no family history of bone disease.

**Figure 1:**
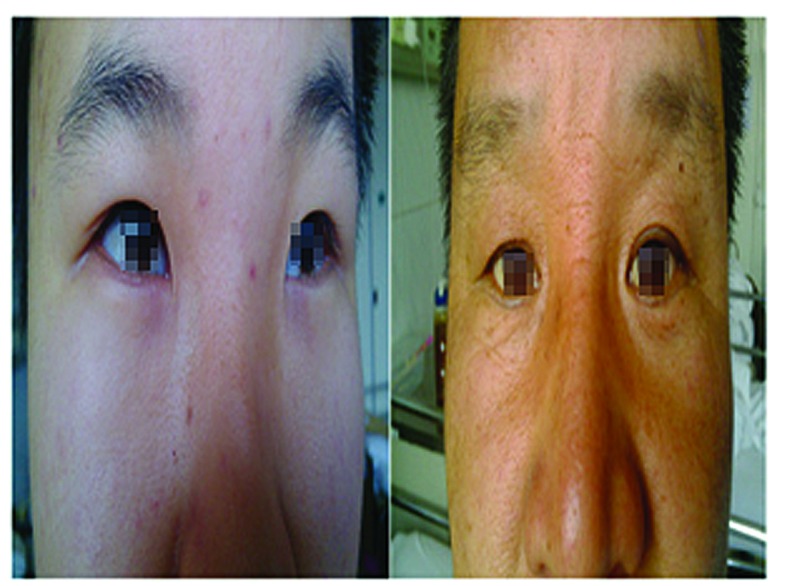
Left image, blue sclera in the patient. Right image, normal sclera in the patient’s father.

**Figure 2:**
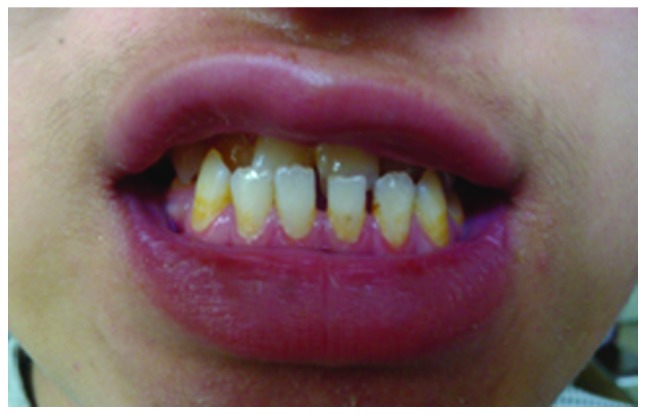
Crossbite of anterior teeth. No discolored or decayed teeth were observed.

**Figure 3:**
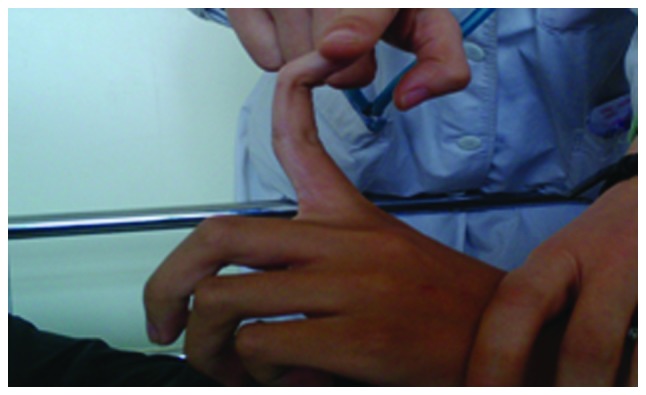
Hypermobility of the joints observed in the patient.

**Figure 4:**
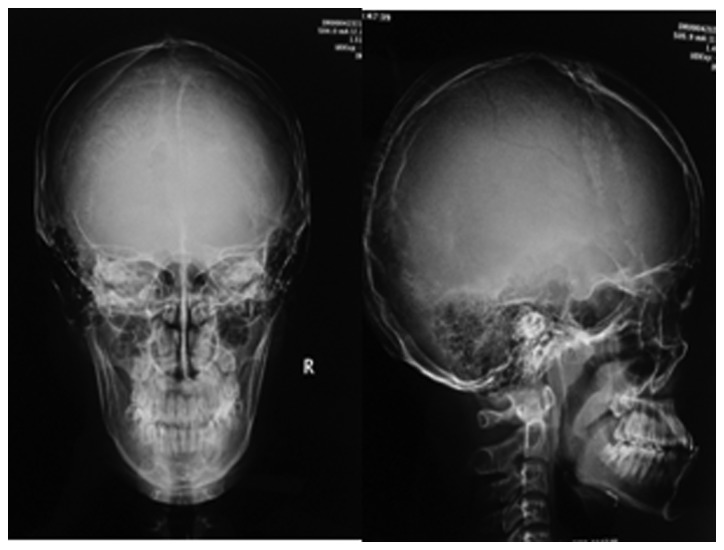
Lateral and frontal X-ray of the skull showed diffuse low bone density.

**Figure 5:**
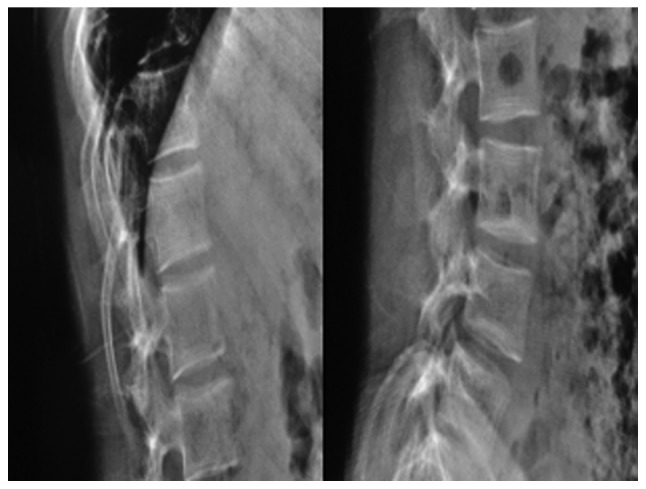
Biconcavity deformities in the lower thoracic and lumbar vertebrae.

**Figure 6:**
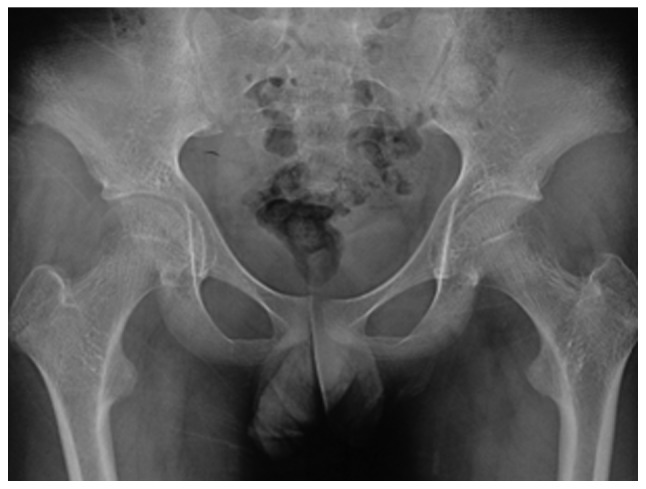
A triradiate pelvis and acetabular protrusion.

The measurements of the patient on admittance to hospital were: Height, 162 cm (25th percentile); body weight, 52 kg (25th percentile) (3); body mass index, 19.8 kg/m^2^; interphalangeal space, 158 cm; and head circumference, 58 cm. On physical examination, the patient displayed normal development, and was well-proportioned with a normal gait and erect posture. The patient’s face was triangular in shape and blue sclera were apparent (Fig. 1). Intraoral examination revealed a crossbite of the anterior teeth and no discolored or decayed teeth (Fig. 2). In addition, altered joint mobility and flexibility were observed (Fig. 3). The patient was estimated to be at stage IV on the Tanner scale of genital and pubic hair development (4). There was no evidence of beading of the ribs, skeletal deformities, hearing impairment, cardiac murmurs or respiratory difficulty and the neurological examination was unremarkable. The laboratory assessments, which included: erythrocyte sedimentation rate; levels of C-reactive protein, serum creatinine, alkaline phosphatase, calcium and phosphorus; and 24-hour urinary excretion of calcium, phosphorus and parathyroid hormone (PTH) all appeared to be within the normal ranges. The serum levels of osteocalcin and total type I collagen telopeptide were above the normal values; however, the level of 25-hydroxyvitamin D was marginally decreased. The levels of thyroid hormone, growth hormone (GH), cortisol, adrenocorticotropic hormone and sex hormones were observed to be within the normal ranges. Protein electrophoresis, rheumatic disease antibody and Bence-Jones protein tests were negative. An X-ray of the skull showed diffuse low bone density (Fig. 4), the absence of Wormian bones and no indication of the ‘salt-and-pepper’ effect. Additional observations obtained from the X-rays included biconcavity deformities in the lower thoracic and lumbar vertebrae (Fig. 5), a triradiate pelvis and acetabular protrusion (Fig. 6), growth arrest recovery lines in the two knee joints and former fractures in the upper end of the fibula. Bone mineral density (BMD) was measured via dual-energy X-ray absorptiometry and low values (mean 0.633 g/cm^2^) for the lumbar vertebra L1–4 were observed. The radiographic features were characteristic of OI and thus led to its diagnosis. A hearing threshold evaluation was conducted and no issues were identified. An electrocardiogram indicated sinus rhythm and an incomplete right bundle branch block. Therefore, the clinical symptoms and radiologic features that were observed resulted in the diagnosis of a mild form of OI type I.

The patient commenced treatment with a regular intake of calcium (1,000 mg/day) in addition to an sufficient intake of vitamin D (800 U/day), these were provided through an ordinary protein-rich diet, extended exposure to sunlight as well as prescriptions of Caltrate and Rocaltrol (calcitriol). The patient was treated with pamidronate infusions intravenously, at a maximum dosage (60 mg) for three days. Following the first infusion, the patient developed an influenza-like reaction, which was accompanied by a rash, fever and a maximum temperature of 38.8°C; the symptoms were controlled using indomethacin and fluid therapy. Following four months of treatment for OI, the symptoms and quality of life of the patient improved and according to the follow-up, the patient continued to take Caltrate and Rocaltrol (one tablet per day), enabling a return to normal schooling, with no bone fractures occurring.

## Discussion

OI, also termed brittle bone disease, is a genetic disorder comprising a heterogeneous group of diseases. It is characterized by a susceptibility to bone fractures with a severity ranging from slight fracture to prenatal fracture. Additional typical clinical features include blue sclera, DI, hyperlaxity of ligaments and skin, hearing impairment, short stature and bone deformities (1). OI presents a complex clinical challenge; it results in abnormal blood coagulation and airway obstruction, cardiovascular anomalies and delayed wound healing (5,6). It is a rare disorder, with an incidence rate of ~1 in 10,000–20,000 births (1).

As OI is a heterogeneous disease and the clinical manifestations are variable, Sillence *et al* divided OI into four types based on their clinical severity and genetic features (7). The classification was as follows: Type I, mild non-deforming; type II, perinatal lethal; type III, severely deforming; and type IV, moderately deforming. However, an increased number of OI cases have subsequently been identified and investigated; therefore, based on the novel clinical, radiological and molecular features, OI types V to VIII have been added to the original Sillence classification (8). These are: Type V, moderate deforming with normal teeth and sclera; type VI, moderate disease with fishscale pattern of bone lamellation, normal sclera and teeth; type VII, which is clinically similar to type II, with the exception that the patients have a smaller head and normal sclera; and type VIII, patients exhibit defects in growth and mineralization (9). OI has been defined as an autosomal dominant disorder resulting from a quantitative or qualitative defect in the synthesis of type I collagen, a predominant and principal extracellular matrix protein within bone tissues (10). The mutation may be in one of the two genes, COL1A1 and COL1A2, which encode for the pro-α1(I) or pro-α2(I) chains of type I collagen (11). However, the resulting phenotypes vary widely, depending on which chains are affected, the position in the collagen structure at which the mutation occurs, and the nature of the amino acid substituent (1). To date, certain forms of OI exhibiting autosomal recessive inheritance have been identified. These genetic causes comprise defects in collagen chaperones and proteins that are involved in type I procollagen assembly, processing and maturation, as well as in proteins that are involved in the formation and homeostasis of bone tissue (12). In the present case, the OI type I was considered to be an autosomal dominant type resulting from a quantitative defect of collagen within normal structures. The clinical diagnosis of OI is predominantly based on the signs and symptoms that are mentioned above, specifically the presence of blue sclera and DI (8). With regards to an etiological diagnosis, collagen analysis and genetic testing is advantageous, but not always necessary. Biopsies from cultured skin fibroblasts may be used to determine the quantity and structure of the type I procollagen molecules, whereas genomic DNA obtained from white blood cells can be screened for mutations (13). However, the association between genotype and phenotype is currently not fully defined. Therefore, a positive result may confirm the diagnosis of OI whereas a negative result does not completely dismiss it (1,8). Thus, the definition of OI is dependent on signs and symptoms.

In the present case, the patient had experienced repeated fractures resulting from minor impacts for three years and routine calcium treatment showed little effect. The physical examination demonstrated blue sclera, hypermobility of the joints, a triangular-shaped face and no hearing impairment or DI. The radiological images revealed osteoporosis and although the clinical features indicated a diagnosis of OI, juvenile idiopathic osteoporosis was also considered to be an alternative diagnosis. The latter disorder is a transient form of childhood osteoporosis without the extraskeletal features that distinguish it from OI. It usually develops in prepubertal, previously healthy infants and persists for 3–5 years without heredity (14).

Once the diagnosis of OI has been established, an evaluation of the patient by a multidisciplinary team is necessary. Physiotherapy, rehabilitation and orthopedic surgery are the mainstays of OI management. The goal of multimodality therapy is to maximize the mobility and functional capabilities of patients (15), and in the present case of mild type I OI, the aim was also to attain a normal quality of life for the patient. Oral and intravenous bisphosphonates (BPs) and potent antiresorptive agents are widely administered for the treatment of all types of OI. Clinical trials have indicated the effectiveness of BPs in the improvement of BMD and the remission of clinical symptoms (10). Although BPs are not a cure for OI, they provide an effective adjunct to comprehensive care. However, optimal usage of BPs, the appropriateness of use by patients with milder symptoms and the side-effects remain unknown. A large scale, randomized double-blind placebo-controlled trial is required to refine their clinical applications. Additional medical therapies are available, such as GHs and PTHs; however, the outcome and adverse effects associated with their use require further assessment and analysis (10).

Surgical intervention is an alternative method of treatment when medical therapies fail. In the present case, no deformities were observed and the reaction to pamidronate was positive; thus, no surgical intervention was required. Additionally, after the patient was discharged from hospital, it was necessary for monitored, moderate physical activity programs to be conducted in order to prevent contractures and immobility-induced bone loss (16). The authors propose psychosocial therapy as a provision for children to address the issues that may arise as a result of repeated bone fractures. In addition, if the patient has children in the future, his partner would require routine prenatal screening, by ultrasound, in addition to genetic counseling since individuals exhibiting a dominant OI trait present a 50% risk of transmission with each pregnancy.

In conclusion, experimental approaches, such as bone marrow and stem cell transplantation, in addition to gene-based therapy, provide potential cures for OI. However, these approaches are currently not ready for clinical trials (10).
